# Fear or food – abundance of red fox in relation to occurrence of lynx and wolf

**DOI:** 10.1038/s41598-017-08927-6

**Published:** 2017-08-22

**Authors:** Camilla Wikenros, Malin Aronsson, Olof Liberg, Anders Jarnemo, Jessica Hansson, Märtha Wallgren, Håkan Sand, Roger Bergström

**Affiliations:** 1 Grimsö Wildlife Research Station, Department of Ecology, Swedish University of Agricultural Sciences, SE-730 91 Riddarhyttan, Sweden; 2 School of Business, Engineering, and Science, Halmstad University, P.O. Box 823, SE-301 18 Halmstad, Sweden; 3 Forestry Research Institute of Sweden, Uppsala Science Park, SE-751 83 Uppsala, Sweden; 4 Gropgränd 2A, SE-753 10 Uppsala, Sweden

## Abstract

Apex predators may affect mesopredators through intraguild predation and/or supply of carrion from their prey, causing a trade-off between avoidance and attractiveness. We used wildlife triangle snow-tracking data to investigate the abundance of red fox (*Vulpes vulpes*) in relation to lynx (*Lynx lynx*) and wolf (*Canis lupus*) occurrence as well as land composition and vole (*Microtus* spp.) density. Data from the Swedish wolf-monitoring system and VHF/GPS-collared wolves were used to study the effect of wolf pack size and time since wolf territory establishment on fox abundance. Bottom-up processes were more influential than top-down effects as the proportion of arable land was the key indicator of fox abundance at the landscape level. At this spatial scale, there was no effect of wolf abundance on fox abundance, whereas lynx abundance had a positive effect. In contrast, at the wolf territory level there was a negative effect of wolves on fox abundance when including detailed information of pack size and time since territory establishment, whereas there was no effect of lynx abundance. This study shows that different apex predator species may affect mesopredator abundance in different ways and that the results may be dependent on the spatiotemporal scale and resolution of the data.

## Introduction

Apex predators may have multiple top-down effects on lower trophic levels in the ecosystem. Classic ecosystem theory predicts that predators control herbivores, restricting their grazing effect on the vegetation and thereby keeping “the world green”^1^. However, the discovery that predators kill, and even consume, not only their herbivore prey, but also members of other competing predator species within their own guild^2–4^, introduced more complexity to ecosystem theory, and supported the Mesopredator Release Hypothesis^5^. According to the Mesopredator Release Hypothesis, the human-caused worldwide reduction of apex predators has not only reduced their impact on herbivores, but also “released” mesopredators from top-down control, allowing them to thrive. The empirical evidence for negative effects of apex predators on mesopredator abundance is strong in both aquatic^6, 7^ and terrestrial systems^8–12^. Although there is ample anecdotal evidence of apex predators killing mesopredators^4, 13^ there are few studies that have been able to demonstrate that this mortality is additive and strong enough to limit mesopredator population growth^14^. It has been suggested that fear of being killed makes mesopredators avoid not only direct proximity to apex predators, but also the habitats used by them which in turn may influence the demography of mesopredator populations^14^.

There may also be reasons for mesopredators to be attracted to apex predators. Most vertebrate carnivores are not only predators, but also facultative scavengers, and scavenging is a prevalent phenomenon in most terrestrial systems^15^. There is an increasing amount of literature on the role and importance of scavenging^15–18^, some even suggesting that scavenging may buffer the effects of climate change^19^. Much of the carrion available to scavengers is provided by apex predators because of their habit of killing large prey, which they often cannot completely consume by themselves^20–24^. Thus, mesopredators are confronted with a dilemma: should they avoid these larger guild members to avoid being killed, even if this means missing scavenging opportunities, or should they take the risk to obtain a free meal? We studied this question in a boreal ecosystem in Sweden where red fox (*Vulpes vulpes*, hereafter fox) was the dominant mesopredator, and Eurasian lynx (*Lynx lynx*, hereafter lynx) and grey wolf (*Canis lupus*, hereafter wolf) were the apex predators.

Both lynx and wolves have been extirpated from most of Europe for several centuries, but recently they have followed a world-wide pattern of large carnivore recovery^25^. The lynx returned to south-central Sweden in the 1950’s after 50 years of absence^26^, and the wolf returned in the 1980’s after having been absent for more than a century^27^. During this time, the fox has remained ubiquitous throughout Europe, including Sweden^28^. Fox abundance and dynamics are linked to the amount of arable land and to the abundance of their main prey, voles (*Microtus* spp.)^28, 29^. For the fox, ungulate remains are also an important food source, especially when accessibility of other food sources is low^30, 31^. When vole abundance is low, foxes may increase their use of ungulate carrion in forest habitats^32^. The consumption of remains from wolf-kills is highest during late winter and spring^33, 34^. Increased food availability during winter has been shown to positively impact the reproductive success of fox^35^. In Sweden, the fox is the most frequent scavenger of wolf-killed moose^34^ (*Alces alces*) and also commonly scavenges remains from lynx-killed roe deer (*C﻿apreolus capreolus*)^36^.

We tested for the two alternative effects of lynx and wolf occurrence on fox abundance at the landscape level (i.e., negative due to higher risk of intra-guild predation or positive due to more scavenging opportunities), while accounting for the known effect of land composition^37^ and vole density^35^. In the next step, we limited our analyses to the wolf territory level and predicted that fox abundance would be negatively related to wolf pack size because more wolves may result in less availability of carrion biomass^38^ and higher predation risk. Finally, we predicted that the negative effect of wolves should be most pronounced during the first years after wolf establishment, because wolves may constitute a novel source of mortality for the fox.

## Results

### Landscape Level

**Figure 1:**
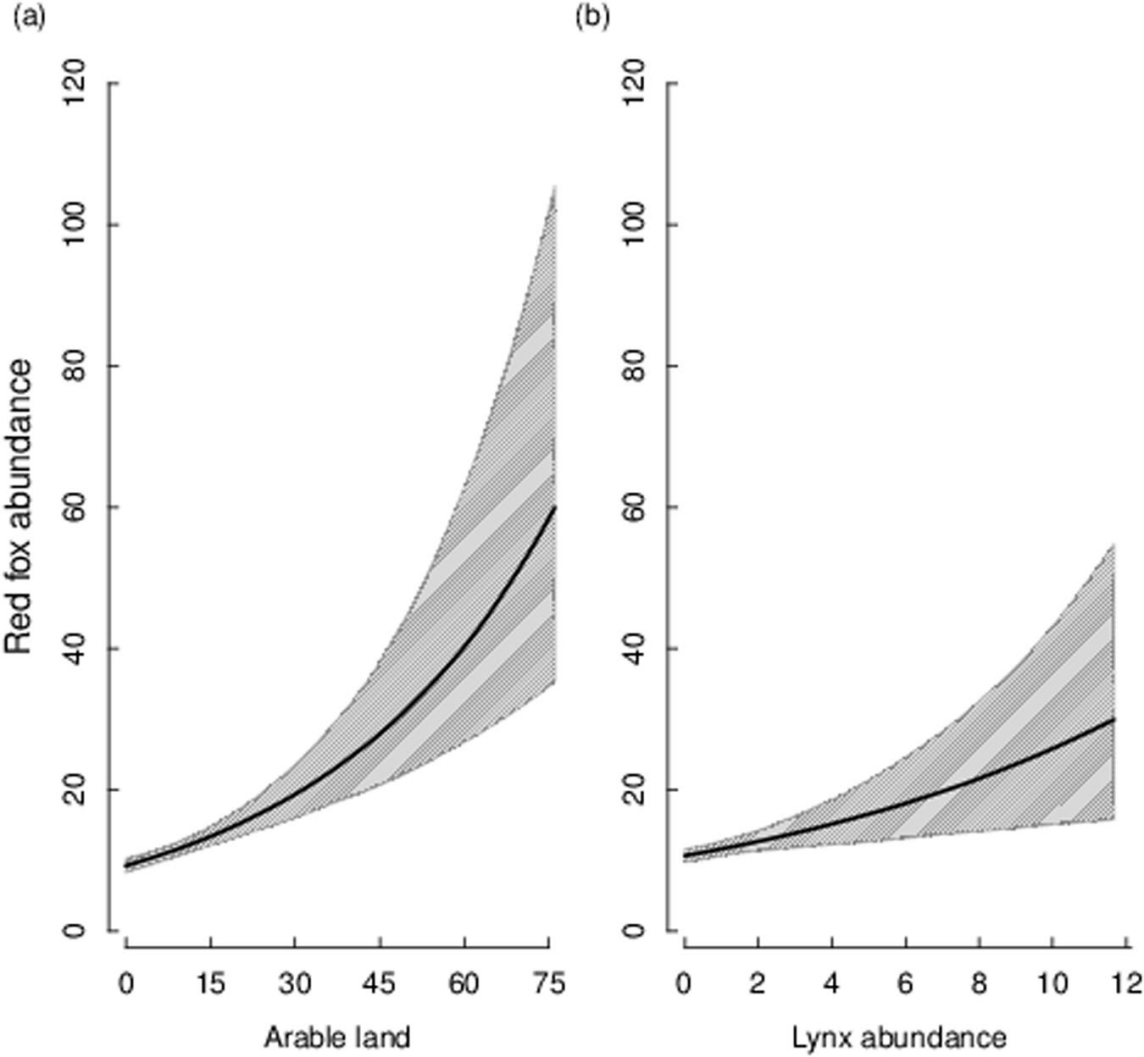
Abundance of red fox (crossings 10 km^−1^ 24 h^−1^) in relation to (**a**) arable land (%) and (**b**) lynx abundance (crossings 10 km^−1^ 24 h^−1^). Model averaged predictions derived from the highest ranked models (Table 1) are shown (solid lines) with associated 95% confidence intervals (grey shading). Predictions for each explanatory variable were made while holding the other variables constant at mean values (arable land 8% and lynx abundance 1.5 crossings 10 km^−1^ 24 h^−1^). Red fox abundance predictions were back-transformed to their normal scale for the figure. For model averaged parameter estimates see Table 2. Red fox and lynx abundance were surveyed by snow-tracking in wildlife triangles in south-central Sweden from 2001–2003.

**Figure 2:**
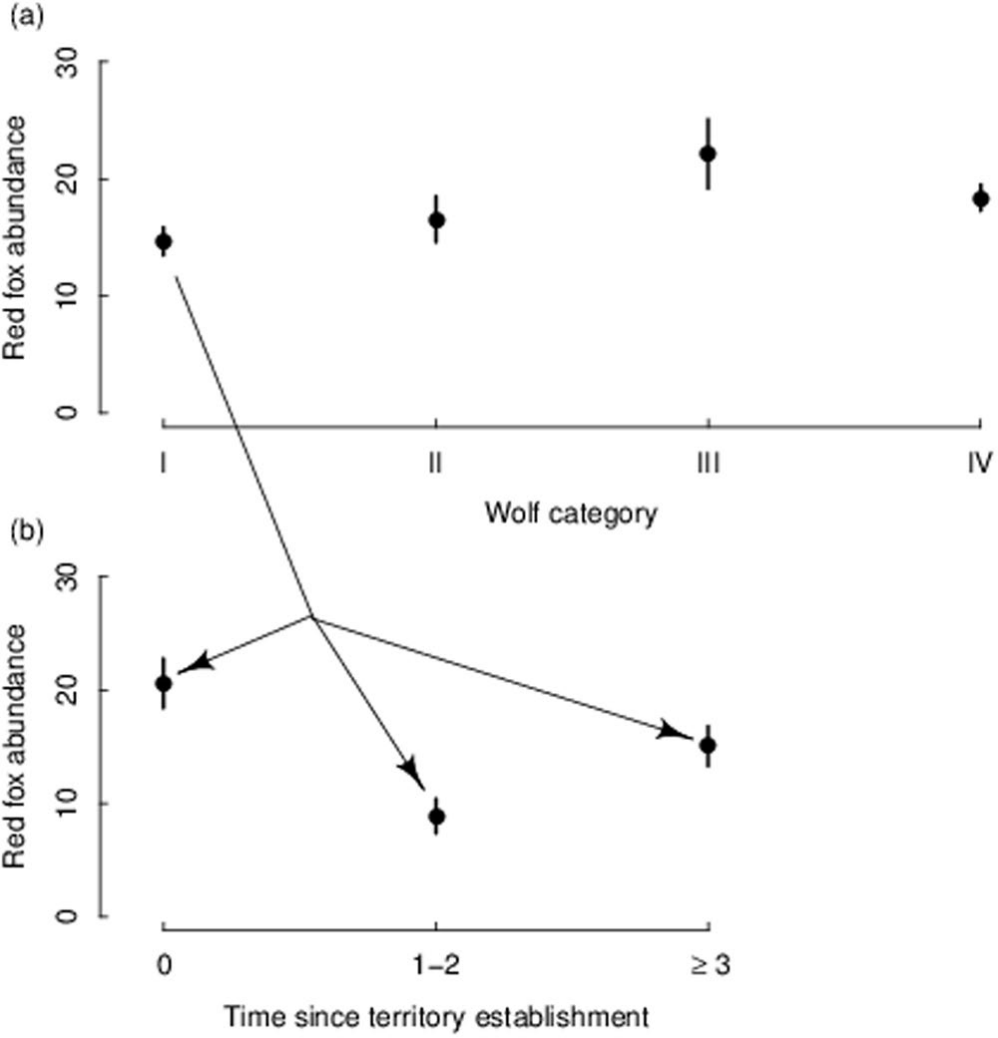
Upper panel (**a**) shows mean (±SE) red fox abundance (crossings 10 km^−1^ 24 h^−1^) in relation to a 4-level wolf category: (I) inside an observed wolf territory, (II) inside an average wolf territory, (III) inside a maximum wolf territory, and (IV) outside a wolf territory. Lower panel (**b**) shows mean (±SE) red fox abundance from wildlife triangles located inside estimated wolf territory borders (i.e., wolf category I) in relation to time since territory establishment (years) where year 0 is the year of territory establishment by a wolf pair. Red fox abundance was surveyed by snow-tracking in wildlife triangles in south-central Sweden fr﻿om 2001–2003.

Fox abundance increased with increasing proportion of arable land, and this variable was the most informative parameter explaining the variation in fox abundance at the landscape level (RVI = 1.0, Fig. 1a and Tables 1 and 2). Fox abundance at the landscape level was also positively related to lynx abundance (RVI = 0.78, Fig. 1b and Tables 1 and 2). There was no effect of wolf occurrence (independent of method used to classify wolf occur﻿rence) on fox abundance (Fig. 2a and Table 1) at the landscape level, nor was there any support for models including the vole density index (Table 1).

**Table 1:** Highest ranked candidate models relating red fox abundance (crossings 10 km^−1^ 24 h^−1^) to arable land (%), vole density index (low or high), lynx abundance (crossings 10 km^−1^ 24 h^−1^), and wolf occurrence.

Wolf occurrence measurement	Model parameters	df	ΔAIC_c_	*w*_*i*_
**1a**. **Wolf abundance**				
	Arable land + Lynx	6	0	0.66
	Arable land	5	2.5	0.19
	Arable land + Lynx + Vole	7	4.7	0.06
	Arable land + Lynx + Wolf abundance	7	5.5	0.04
	Arable land + Vole	6	7.2	0.02
	Lynx	5	21.4	<0.01
	Intercept only	4	26.4	<0.01
	Vole	5	31.4	<0.01
	Wolf abundance	5	32.7	<0.01
**1b**. **Wolf category**				
	Arable land + Lynx	6	0	0.69
	Arable land	5	2.5	0.20
	Arable land + Lynx + Vole	7	4.7	0.07
	Arable land + Vole	6	7.2	0.02
	Arable land + Lynx + Wolf category	9	7.5	0.02
	Lynx	5	21.4	<0.01
	Intercept only	4	26.4	<0.01
	Vole	5	31.4	<0.01
	Wolf category	7	36.6	<0.01
**2**. **Wolf pack size**				
	Vole	5	0.0	0.21
	Arable land + Vole	6	0.5	0.16
	Arable land	5	0.9	0.13
	Arable land + Wolf pack size	6	0.9	0.13
	Intercept only	4	1.1	0.12
	Arable land + Vole + Wolf pack size	7	2.0	0.08
	Vole + Lynx	6	4.1	0.03
	Wolf pack size	5	4.2	0.03
	Vole + Wolf pack size	6	4.7	0.02
	Lynx	5	5.0	0.02
	Arable land × Wolf pack size	7	5.1	0.02
	Arable land + Vole + Lynx	7	5.2	0.02
**3**. **Time since territory establishment**				
	Time	6	0.0	0.16
	Arable land + Time	7	0.1	0.15
	Vole	5	0.4	0.13
	Time + Vole	7	0.7	0.11
	Arable land + Vole	6	0.9	0.10
	Arable land + Time + Vole	8	0.9	0.10
	Arable land	5	1.4	0.08
	Intercept only	4	1.5	0.07
	Time + Lynx	7	4.3	0.02
	Vole + Lynx	6	4.5	0.02
	Lynx	5	5.4	0.01

We used fo﻿ur separate measurements of wolf occurrence: 1a) *wolf abundance* (crossings 10 km^−1^ 24 h^−1^); 1b) a four-level *wolf category* (I: inside an observed wolf territory, II: inside an average wolf territory, III: inside a maximum wolf territory, or IV: outside a wolf territory); 2) *wolf pack size* (range 2–8 individuals), using only triangles inside wolf category I; and 3) *time since territory establishment* (year of territory establishment, 1–2 years after establishment, or ≥3 years after establishment), using only triangles inside wolf category I. Wildlife triangle identity and year of survey were used as random effects to account for repeated measurements and year effects. For each model, we show degrees of freedom (df), difference in AIC_c_ relative to the highest-ranked model (ΔAIC_c_), and AIC-weight (*w*_*i*_). For simplicity, only models with *w*_*i*_ > 0.01, univariate models, and intercept-only model are shown. Red fox, lynx, and wolf abundances were surveyed by snow-tracking in wildlife triangles in south-central Sweden from 2001–2003. Wolves were also monitored in a national monitoring program and voles were monitored using snap traps during long-term monitoring at Grimsö Wildlife Research Area.

**Table 2:** Model-averaged parameter estimates with standard error (SE) for each variable retained in the best models (ΔAIC_c_ ≤ 2) in Table 1.

Wolf occurrence measurement	Model parameters	Estimate	SE
***1a***. **Wolf abundance; ﻿﻿1﻿b.﻿ Wolf category**			
	Intercept	2.17	0.060
	Arable land	0.025	0.0042
	Lynx abundance	0.089	0.028
**2**. **Wolf pack size**			
	Intercept	2.22	0.29
	Arable land	0.053	0.018
	Vole	−0.33	0.15
	Wolf pack size	−0.028	0.011
**3**. **Time since territory establishment**			
	Intercept	2.47	0.28
	Arable land	0.038	0.013
	Vole	−0.26	0.12
	Time	1–2: −0.44	1–2: 0.15
		≥3: −0.25	≥3: 0.13

We used fo﻿ur separate measurement of wolf occurrence, as defined in Table 1. Vole density index is a two-category variable where the parameter estimate is the difference in red fox abundance for low compared to high (intercept) vole density index. Time since territory establishment class is categorical, where the parameter estimate is the difference in red fox abundance at 1–2 years or ≥3 years after territory establishment compared to the year of establishment (intercept).

### Wolf Territory Level

**Figure 3:**
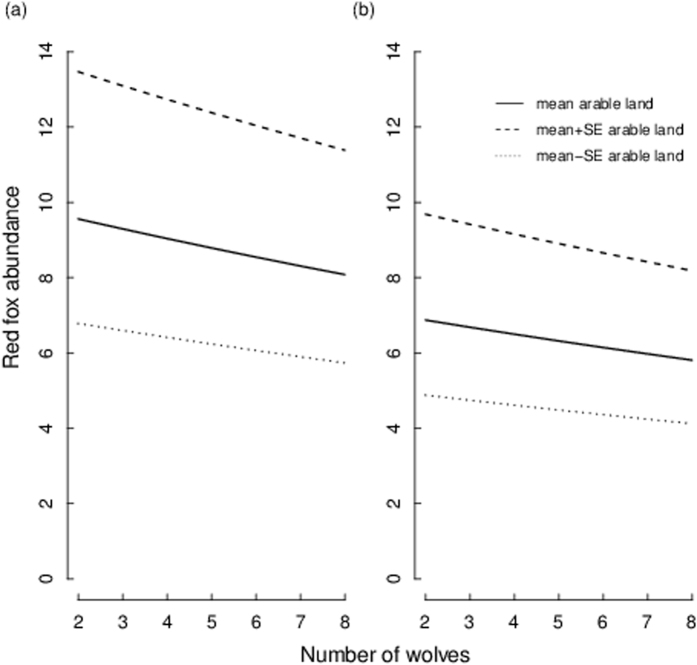
Red fox abundance (crossings 10 km^−1^ 24 h^−1^) in relation to wolf pack size, arable land (%) and vole density index (low or high). Model averaged predictions derived from the highest ranked models (shown in Table 1) are shown for different wolf pack sizes at (**a**) high and (**b**) low vole density index, while holding the arable land constant at the mean value (2% within wolf territory borders, solid line). Dashed lines indicate red fox abundance when arable land varies with ±1 SE (±0.3). Red fox abundance predictions were back-transformed to their normal scale for the figure. Red fox abundance was surveyed by snow-tracking in wildlife triangles in south-central Sweden from 2001–2003.

At the wolf territory level there was a weak negative relationship between fox abundance and wolf pack size (RVI = 0.29) in combination with the proportion of arable land, although arable land and vole density index were the best predictors of fox abundance (RVI = 0.56 and 0.53, respectively, Fig. 3 and Tables 1 and 2). Including time since territory establishment in the analyses showed that there was a negative temporal effect of wolves on fox abundance (RVI: time since territory establishment = 0.57, vole density index = 0.49, arable land = 0.47). Fox abundance decreased 1–2 years after wolf territory establishment and after these first two years fox abundance rebounded again, although to a slightly lower level than at the year of wolf territory establishment (Fig. 2b). In contrast to the landscape level, there was no relationship between fox abundance and lynx abundance at the wolf territory level (Tables 1 and 2).

## Discussion

Although both wolves and lynx had a top-down effect on fox abundance in our study area during winter, bottom-up processes were more influential, as the proportion of arable land was the key indicator of fox abundance. The almost complete loss of apex predators during the 19^th^ century, in combination with geographical variation in ecosystem productivity, has been shown to be a key factor in the fox population increase in Scandinavia^8^. The agricultural landscape holds the highest densities of foxes^28^. Also, lynx in Scandinavia are attracted to agricultural fields because their main prey, roe deer, are clumped around this habitat^39–41^. Wolf territories, on the other hand, are composed by a small amount of open areas, such as agricultural fields, suggesting an adaptation to avoid humans^42^. Thus, in our study area, fox and lynx likely spatially overlap more than fox and wolves. The avoidance of open areas by wolves is further supported by the fact that the proportion of arable land in wildlife triangles inside wolf territories averaged 2% (range 0–20%), compared to 8% (range 0–76%) in the whole study area. The importance of vole density on fox abundance may be more pronounced in less favorable habitats^29^. Thus, the lower proportion of arable land within wolf territories may explain why the vole density index affected fox abundance at the wolf territory level, but not at the landscape level. Consequently, the lowest fox abundance was inside wolf territories that had a low proportion of arable land and low vole density index. This supports the conclusion that bottom-up processes were an important factor affecting fox abundance.

At the landscape level, there was a positive relationship between fox and lynx abundance. Simulations where we added observation uncertainty to the lynx abundance measurements (i.e., coefficient of variation ranging from 0.2 to 3) revealed that our results were robust; it was a high probability (i.e., ˃97%) of a positive relationship between fox abundance and lynx abundance (see Supplementary Methods for further details). This result was surprising since several studies have shown a negative relationship between these two species^8, 9, 28, 43^. For example, Elmhagen & Rushton^8^ used historic data to demonstrate a mesopredator release of fox after lynx and wolves were exterminated from Sweden, indicating that foxes had been top-down limited before the extermination of these two apex predators, and Elmhagen *et al*.^9^ found that this process was reversed when lynx recolonized parts of Finland in recent time. Our data did not allow us to determine whether the positive effect of lynx abundance on fox abundance in our study was a demographic or a behavioral response. Although previous studies have suggested a negative demographic effect of lynx on fox populations^8, 9, 13^, we suggest that the effect we observed was a behavioral response by fox to lynx abundance. Foxes are attracted to sites with olfactory cues from lynx, a behavior that should increase the possibility to access carrion from lynx killed prey^44^. However, from lynx scent marks, a fox might also gain information about the existence of lynx in its home range, a knowledge that may be used to adjust behavior in order to reduce the risk of encountering lynx^44^. Studies of multiple predator sympatry in African and Asian ecosystems have shown fine-scale behavioral tactics and trade-offs associated with resource availability^45–47^. Hence, we suggest that the positive relation between fox and lynx abundance indicates that foxes are attracted to lynx to increase their chances to find carrion, possibly facilitated by using a fine-scale spatiotemporal segregation strategy to minimize the risk of being killed. Two alternative hypotheses are that 1) both lynx and fox are attracted to a third, currently unknown, factor, or 2) species within the same mammalian family are likely to compete more than species in different families^48^, which causes fox to avoid wolves and not lynx. In any case, our findings contradict the hypothesis that the avoidance of apex predators could suppress mesopredator abundance more than predator-induced mortality^14^, at least with respect to fox and lynx in our study area.

Whereas we found no effect of wolves on fox abundance at the landscape level, we show a negative temporal effect of wolf occurrence at the wolf territory level. This effect was most pronounced the first two years immediately after wolf territory establishment. We suggest that this negative effect is explained by a combination of the novelty of wolf occurrence and their local abundance, i.e., pack size. Zimmermann *et al*.^38^ showed that large wolf packs (6–9 wolves) in Scandinavia had a kill rate that provided just enough, or even less, prey biomass than required, while small packs (2–3 wolves) had a kill rate that provided more prey biomass than required, thereby leaving a larger surplus of carrion. Furthermore, the probability that foxes encountered wolves increased in large pack territories due to the tendency of large wolf packs to split up in sub-groups during winter^49^. The establishment of a wolf territory usually starts with a breeding pair, followed by an increased number of wolves the consecutive winter after the pair’s first reproduction. Fox abundance one and two years after wolf establishment was reduced by 50% compared to the establishment year and with triangles outside wolf territories (wolf category III and IV in Fig. 2a). The reason that we did not see any negative or positive effect during the establishment year could be either that the pack consisted only of the establishing pair, i.e., the smallest possible pack size, and/or that the pair was formed late in the winter. Consequently, we suggest that the pronounced negative effect on fox abundance the years immediately following wolf establishment was due to an avoidance reaction by foxes to the novelty of wolf presence which faded over time, or the increased mortality of naïve foxes. We suggest that a demographic effect is less likely than a behavioral effect as wolf scat analyses indicate that intra-guild predation by wolves on foxes is low in Scandinavia (<0.2% of wolf scats (n = 2091) contained hair from fox)^50, 51^. However, if wolves kill foxes primarily to reduce competition (i.e., foxes are not consumed), wolf scat analysis might underestimate intra-guild predation. But studies of wolf predation in Scandinavia show that few wolf-killed foxes are found nearby the remains of wolf-killed ungulates^52, 53^ which also indicates low intra-guild predation.

We argue that food competition is not a plausible cause for the negative effect of wolves on fox abundance. Wolf occurrence results in increased carrion availability, a food source frequently utilized by foxes in our study area^34^. Although wolves occasionally kill smaller prey species (hares (*Lepus timidus*, *Lepus europeus*) and forest grouse (*Tetrao tetrix*, *Tetrao urogallus*))^53^, that are also prey for foxes, wolf predation on these species is likely not high enough to affect their abundance. After three or more years since the wolf territory was established, fox abundance rebounded. Unfortunately, our data set did not allow us to assess whether or when fox abundance rebounded starting at pre-wolf numbers. We were unable to conduct a similar analysis of possible time effects on the response of foxes to lynx, since lynx had already established in the area several years before our study started^13^. It is possible that the fox population underwent a learning process, comparable to what appeared to have occurred with wolves, which enabled them to co-exist with lynx. This process is likely more advanced with the lynx population when compared to the wolf populations, due to the longer time of co-existence between fox and lynx in our study area.

In our study, foxes were positively affected by lynx abundan﻿ce, while wolf occurrence had a negative temporal effect on fox abundance, although support for the latter effect was weak. The observed response of foxes to these two apex predator species contradicts the results from Pasanen-Mortensen *et al*.^43^ and Pasanen-Mortensen & Elmhagen^28^, who found a strong negative effect of lynx, and no effect of wolves, on fox abundance at the Eurasian scale. This is even more puzzling considering wolves, which prey mainly on moose, likely leave more carrion than lynx, which mainly rely on smaller roe deer^34, 41^. Also, because the risk of intra-guild predation is greater when two species are of similar size, there should be greater risk for foxes in lynx areas than in wolf areas^48^. However, the extent of top-down control may depend on the density of the apex predators^11, 54^. Unfortunately, a comparison between apex predator density in our small-scaled, short-term study (stable or slightly decreasing lynx population and a recolonizing wolf population) and previous studies conducted in Scandinavia is not straightforward due to different measurements of apex predators occurrence (presence/absence^28, 43^, harvest statistics^8﻿^), and variation in spatial scales (country^8, 9^, continent^28, 43﻿^), time span (long-term^9﻿^), and population phase (recolonized lynx population^9﻿^). Our study suggests that the relationship between fox, lynx and wolves might be more complex than observed at a larger scale, or by only using presence/absence data. Actually, Guillaumet *et al*.^55^ found that by reducing the spatial scale of analysis there was a positive synchrony between Canadian lynx and coyote (*Canis latrans*) numbers, contrary to the negative pattern observed at a larger spatial scale. Furthermore, temporal partitioning of activity may facilitate the coexistence of foxes and apex predators^56^. The importance of spatiotemporal scale is further illustrated by our results that at a larger spatial scale (the landscape level) fox abundance and wolf occurrence were not correlated (c.f., Pasanen-Mortensen *et al*.^43^), whereas at a smaller spatial scale (the wolf territory level) we found a negative temporal effect of wolf occurrence on fox abundance. Our results emphasize the importance of considering multiple spatiotemporal scales when investigating species interactions. Further studies at an even smaller scale than used in our study (e.g., locations of tracks in space and time within wildlife triangles or using GPS-collared individuals) are warranted to gain further insight into the impact of lynx and wolf on fox, and determine how this is influenced by habitat.

## Materials and Methods

### Study System

The study was conducted from 2001–2003 in the south-central part of Sweden within the counties of Värmland and Örebro (referred to as the landscape level, 58°50′–60°N, 12°–17°E, approximately 26 000 km^2^). The area is dominated by boreal forest interspersed with numerous lakes, rivers and mires that contribute to a more variable landscape than is found in other areas within the boreal forest zone. The proportion of arable land increases from north-northeast to the south-southwest. About 74% of the total land area of Värmland, and 67% of Örebro, is forested and the corresponding area of arable land is 6% and 16%^57^. The number of days with snow ranges from approximately 75/year in the south to approximately 175/year in the north^58^. For a detailed description of the study area see Wallgren *et al*.^59^.

In the 1980s, the density of the fox population decreased dramatically due to an epizootic outbreak of sarcoptic mange^60^, and subsequently recovered in the 1990s^61^. Lynx reoccupied south-central Sweden in the beginning of the 1950s^26^ and, by 2002–2003, the total Swedish lynx population was estimated to 1300–1600 individuals^62^. During the study period the number of lynx family groups (i.e., female with kittens, the monitoring unit within the national lynx monitoring program)^62^ in the study area was 69 (2001) and 49–55 (2003). Roe deer are the main prey for lynx within the study area^41^. Wolves returned to the study area in the early 1980s through natural re-colonization and, in 2002–2003, the total Swedish wolf population was estimated at 84–100 individuals^63^. During the study period (winters of 2000/2001–2002/2003), the study area had 8, 10, and 10 wolf territories, including scent marking pairs or family groups (≥3 wolves)^63–65^. Moose are the main prey for wolves in Scandinavia throughout the year^38, 53^.

### Wildlife Triangles and Classification of Wolf Territories

**Figure 4:**
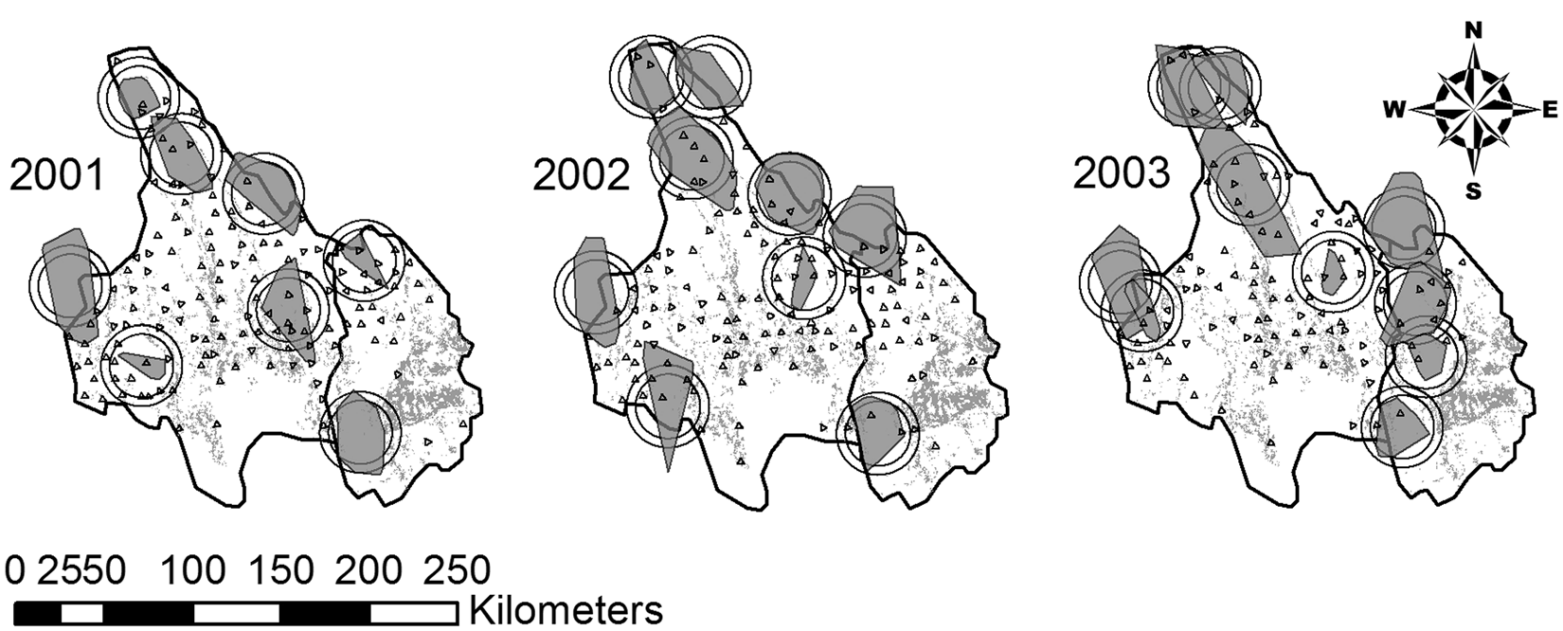
Location of wildlife triangles in south-central Sweden (county of Värmland to the left and county of Örebro to the right) where red fox, lynx, and wolf abundances were surveyed by snow-tracking from 2001–2003, in relation to arable land (grayed, data source land use: 2.1 Arable land and 2.3 Pastures, Corine Land Cover (CLC) 2006, version 18.5 (http://www.eea.europa.eu/data-and-maps/data/clc-2006-raster)). Triangles were classified according to a 4-level wolf category: (I) inside an observed wolf territory (grey polygons according to monitoring data^63–65﻿^), (II) inside an average wolf territory (smallest circles), (III) inside a maximum wolf territory (largest circles), and (IV) outside a wolf territory (if not included in category I–III). Maps created using ArcGIS 10.4 (http://support.esri.com/Products/Desktop/arcgis-desktop/arcmap/10–4).

Snow-tracking data on fox, lynx, and wolves were collected between January 15 and March 20, 2001–2003, along 182 equilateral triangular routes^66^. The triangles had a side length of 4 km and were regularly spaced at predetermined positions about 10 km apart. Intersecting tracks were recorded during 1–4 visits per triangle each winter, each triangle visit occurred ≤4 days (mean 2 days) after snowfall (n_2001_ = 205, n_2002_ = 184, n_2003_ = 106 triangle visits) resulting in a total of 495 triangle visits during the three study years (all triangles were not visited all years, Fig. 4). For each triangle visit, we calculated the relative abundance of each species as crossings 10 km^−1^ 24 h^−1^, following Kurki *et al*.^37^. Kurki *et al*.^37^ demonstrated that abundance based on snow-tracking was significantly correlated with hunting bags of two mesopredators (fox and pine marten (*Martes martes*)) although other factors also contribute to variation in bag. The use of snow-tracking as an index of abundance is further exemplified by Lyly *et al*.^67^, but may not work for all species or areas^68^.

Fox tracks were recorded at almost all triangle visits, i.e., during 493 of the 495 visits (mean = 15 ± 0.03 SE crossings 10 km^−1^ 24 h^−1^, range 0.2–139) while lynx tracks were found during 172 visits (mean = 1.5 ± 0.01 SE crossings 10 km^−1^ 24 h^−1^, range 0.2–12). Wolf tracks were only recorded in 49 triangle visits (mean = 1.9 ± 0.05 SE crossings 10 km^−1^ 24 h^−1^, range 0.2–15). To analyse the potential effect of wolves on fox abundance, we used two different data sets: (a) wolf abundance (crossings 10 km^−1^ 24 h^−1^) from the wildlife triangles as described above, and (b) wolf category (I–IV, see below) based on the distance between the triangle and nearest wolf territory with scent-marking wolf pairs or family groups (Fig. 4), available from the national wolf-monitoring system conducted annually by the County Administrative Boards^49^. Wolf territory borders were estimated based on the monitoring data^63–65^ and locations from VHF- or GPS-collared wolves (available in 22 annual territories). All procedures including capture, handling, and collaring of wolves^69^ was in accordance with ethical requirements and approved by the Swedish Animal Welfare Agency. This dataset also includes estimates of the minimum number of wolves per wolf territory (range: 2–8 individuals) and time since territory establishment (range: 0–22 years where 0 equals the year of wolf establishment).

As available data from snow-tracking and locations from VHF-collared wolves (two locations per week on average) did not give a full representation of wolf territory borders, we measured distances between the centre of each wildlife triangle to the centre of the nearest wolf territory (using ArcView 3.0, ESRI Corporation, Redlands, CA) and classified wolf occurrence for each wildlife triangle according to the following four categories: (I) *inside an observed wolf territory* if the triangle centre was located within the confirmed borders of a wolf territory, (II) *inside an average wolf territory* if the triangle centre was not located inside an observed wolf territory but within a distance corresponding to the average wolf territory radius from the nearest territory centre (18.0 km, average wolf territory size = 1017 km^2^)^70^, (III) *inside a maximum wolf territory* if the triangle centre was not located inside an observed or an average wolf territory but within a distance corresponding to the maximum wolf territory radius from the nearest territory centre (23.0 km, maximum wolf territory size = 1676 km^2^)^70^, or (IV) *outside a wolf territory* if the distance from nearest territory centre was longer than the radius used in category III. Thus, a triangle belongs to only one category.

### Habitat Composition and Vole Density Index

As arable and pasture land are known to have a positive impact on fox density due to food abundance^8, 37^, we calculated the percentage of these habitats (pooled and referred to as arable land), using the Swedish Land Cover Data^71^ (similar to the European Union classification system Corine Land Cover (CLC)). This was done within a 1 km buffer zone around each wildlife triangle (mean = 8%, range: 0–76%) in order to account for the effect of land composition on fox abundance (Fig. 4). For a detailed description of the methodology and classification see Wallgren *et al*.^59^. Furthermore, the fox population tends to fluctuate following the variation in vole density with a time lag of one year^35, 72^. To account for the effect of vole density, we used the annual estimates of vole density index from Grimsö Wildlife Research Area (located within the study area; 59°40′N, 15°25′E), where bank vole (*Clethrionomys Glareolus*) and field vole (*Microtus agrestis*) have been monitored by capture in snap traps in May each year since 1973. In years 2000, 2001, and 2002, the vole density index (both species pooled) was 0.50, 0.49, and 0.81 voles captured per 100 trap nights^73^. During the period 1990–2010, the vole density index varied between 0 and 1.51. Consequently, the vole density index was classified as low in 2001 and 2002 and high in 2003 for our study period.

### Statistical Analyses

We conducted all statistical analyses in R version 3.2.2^74^ using General Linear Mixed Models in the *lme4* package^75^ with fox abundance (crossings 10 km^−1^ 24 h^−1^) as a response variable. Since fox abundance is a continuous variable, and its variance increased with increasing mean (i.e., similar to a log-normal distribution where σ = μ^2^), we used log-transformed fox abundance and a Gaussian error distribution in all models to fulfil the assumptions of linearity. We used wildlife triangle identity and year as random effects in all models to account for repeated measurements and year effects. To test the effect of apex predators on fox abundance at the landscape level, we included four explanatory variables: wolves (classified according to wolf abundance or wolf category, as described in (a) and (b) above, exclusive of each other), lynx abundance (crossings 10 km^−1^ 24 h^−1^), proportion of arable land (%), and vole density index (2-level categorical: low or high). We also included pairwise interactions between habitat and all other explanatory variables as well as an interaction between wolf and lynx abundance.

To assess the effect of wolf pack size and time since territory establishment on fox abundance, we restricted our analyses to data from wildlife triangles that were exclusively inside the observed borders of a confirmed wolf territory (wolf category I, n = 77, referred to as the wolf territory level). We used wolf pack size and time since territory establishment (3-level categorical: year of establishment (referred to as 0), 1–2 years after establishment, or ≥3 years after establishment) together with lynx abundance, proportion of arable land, and vole density index as explanatory variables in the models.

For each analysis, we compared candidate models using the sample-size corrected Akaike Information Criterion (AIC_c_) and AIC weights (*w*_*i*_) from the ‘*MuMIn*’ package^76^ in R. Models with ΔAIC_c_ <2 were used to generate model-averaged parameter estimates^77^. We used a bootstrap method implemented in R using the *‘ez’* package^78^ to calculate predictions and 95% confidence intervals for mixed models. Additionally, we used AIC_c_ weights on the full candidate model set to generate Relative Variable Importance weights (RVI) for each explanatory variable.

### Data Availability

All data analysed during this study are included in the Supplementary Data file.

## Electronic supplementary material

Supplementary Methods

Supplementary Data
