# Supplementary material for: Fear or food – abundance of red fox in relation to occurrence of lynx and wolf
Source: Sci Rep. 2017 Aug 22;7:9059. doi: 10.1038/s41598-017-08927-6 (PMC5567382; doi:10.1038/s41598-017-08927-6)
Supplement: Supplementary file 1 — Supplementary Methods [file 41598_2017_8927_MOESM1_ESM.pdf]

Supplementary Methods

Fear or food – abundance of red fox in relation to occurrence of lynx and wolf

Camilla Wikenros[1], Malin Aronsson[1], Olof Liberg[1], Anders Jarnemo[2], Jessica Hansson[2], Märtha Wallgren[3], Håkan Sand[1], and Roger Bergström[3,4]

[1]Grimsö Wildlife Research Station, Department of Ecology, Swedish University of Agricultural Sciences, SE-730 91 Riddarhyttan, Sweden
[2]School of Business, Engineering, and Science, Halmstad University, P.O. Box 823, SE-301 18 Halmstad, Sweden
[3]Forestry Research Institute of Sweden, Uppsala Science Park, SE-751 83 Uppsala, Sweden. [4]Present address: Gropgränd 2A, SE-753 10 Uppsala, Sweden

Correspondence and requests for materials should be addressed to C.W. (email: camilla.wikenros@slu.se)

Abundance data based on track-crossings is likely to include uncertainties due to observation error. We used a Bayesian approach to assess how our results are influenced by observation errors in explanatory variables used in the analysis, following Hobbs *et al.* [79].

Fox abundance was influenced by the index for lynx abundance, but not wolf abundance (see Table 1 of the main text). Consequently, we used the explanatory variables included in the best model in Table 1 in the corresponding Baysian model:

$$\mu_i = \alpha_{j[i]} + \beta_1 \text{Lynx}_i + \beta_2 \text{Arable land}_i$$
$$y_i \sim \text{normal}(\mu_i, \sigma)$$

where μ is the deterministic prediction of fox abundance on the log scale, y the fox abundance measurement on the log scale, β_1 is the regression coefficient for lynx, β_2 is the regression coefficient for arable land, and the subscript i an index fox abundance measurements. Triangle identity was included as a group level effect on the intercept, indexed by subscript j:

$$\alpha_j \sim \text{normal}(\mu_\alpha, \sigma_\alpha)$$

We used a Bayesian Gibb's sampler (i.e., JAGS [80]) called from R using the 'rjags' package [81] to estimate final model parameters and generate predictions. For each model, we ran two independent chains with different starting values. Convergence was assessed using the Gelman and Rubin diagnostic [82] (<1.1). Model parameters from the Bayesian model were very similar to the parameters from the General Linear Mixed Model in Table 2 of the main text (i.e., log (fox abundance) = 2.17 (± 0.055 SE) + 0.025 (± 0.0042 SE) Lynx + 0.088 (± 0.028 SE) Arable land).

To assess the consequence of uncertainty in lynx abundance, we assumed each lynx abundance measurement was associated with a standard deviation (σ_{obs}) equal to the abundance measurement (Lynx_{obs}) multiplied by coefficients of variation ranging from 0.2 to 3.0, and that the "true" lynx abundance (Lynx_{true}) was distributed as:

$$\text{Lynx}_{true} \sim \text{gamma}\left(\frac{\text{Lynx}_{obs}^2}{\sigma_{obs}^2}, \frac{\text{Lynx}_{obs}}{\sigma_{obs}^2}\right)$$

For each coefficient of variation, we estimated the probability that the lynx coefficient in the model was positive by calculating the proportion of the posterior distribution of the coefficient that was above zero ($P(\beta_1>0)$). Our simulations indicate that our overall conclusion of a positive relationship between fox abundance and lynx abundance was robust, as there

was a high probability for positive β_1 for coefficient of variation ≤3 (Supplementary Figure 1). In fact, the lower 95% credible interval for β_1 was <0 first at coefficient of variation = 2.5 and, even though the uncertainty around the parameter estimate increased with increasing coefficient of variation, the probability of obtaining a negative relationship between fox and lynx abundance was ≤2.7% for coefficient of variation ≤3.

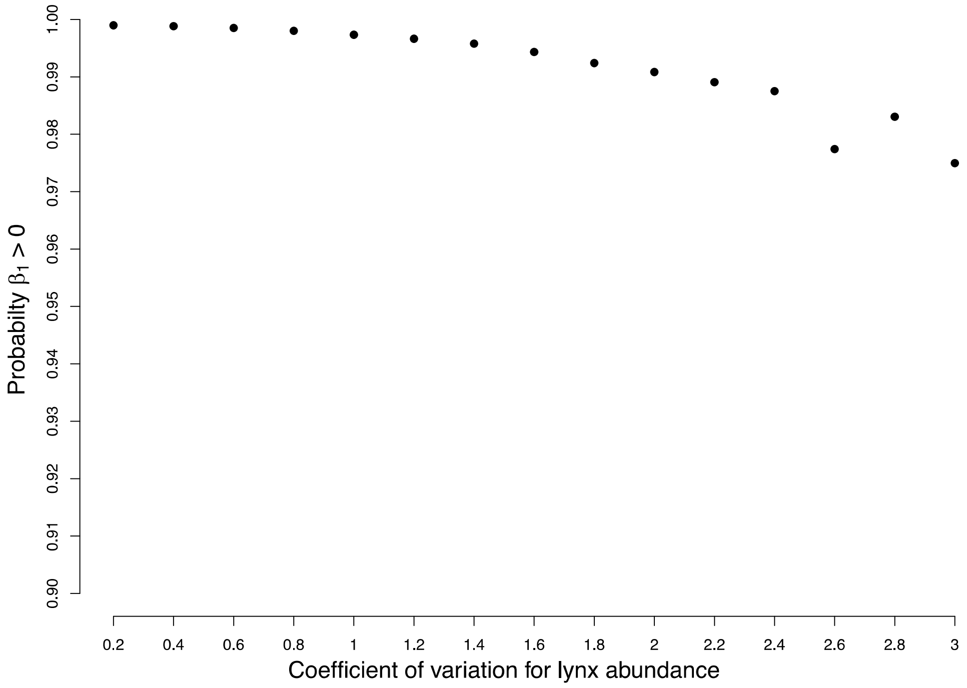

Supplementary Figure 1. Simulations of the effect of observation error in the conclusion that lynx abundance positively influenced fox abundance in our study. The x-axis shows the assumed coefficient of variation for lynx abundance and the y-axis shows the proportion of the estimates in the MCMC chain for which the estimate for the model coefficient for lynx (β_1) was above zero.

References

79. Hobbs, N.T., Andrén, H., Persson, J., Aronsson, M. & Chapron, G. Native predators reduce harvest of reindeer by Sámi pastoralists. *Ecol. Appl.* **22**, 1640-1654 (2012).
80. Plummer, M. JAGS: a program for analysis of Bayesian graphical models using Gibbs sampling. R Foundation for Statistical Computing, Vienna, Austria (2003).
81. Plummer, M. rjags: Bayesian graphical models using MCMC. R package version 3-13. http://CRAN.R-pro- ject.org/package=rjags (2014).
82. Gelman A. & Rubin D.B. Inference from iterative simulation using multiple sequences. *Statist. Sci.* **7**, 457-511 (1992).
